# High-resolution MEMS inertial sensor combining large-displacement buckling behaviour with integrated capacitive readout

**DOI:** 10.1038/s41378-019-0105-y

**Published:** 2019-12-16

**Authors:** Brahim El Mansouri, Luke M. Middelburg, René H. Poelma, Guo Qi Zhang, Henk W. van Zeijl, Jia Wei, Hui Jiang, Johan G. Vogel, Willem D. van Driel

**Affiliations:** 1 Electronic Components, Technology and Materials, Faculty of Electrical Engineering, Delft University of Technology, Mekelweg 4, 2628CD Delft, The Netherlands; 2 Else Kooi Laboratory, Feldmannweg 17, 2628CT Delft, The Netherlands; 3 Electronic Instrumentation, Faculty of Electrical Engineering, Delft University of Technology, Mekelweg 4, 2628CD Delft, The Netherlands; 4 Signify, High Tech Campus 48, 5656AE Eindhoven, The Netherlands

**Keywords:** Electrical and electronic engineering, Engineering, Nanoscience and technology

## Abstract

Commercially available gravimeters and seismometers can be used for measuring Earth’s acceleration at resolution levels in the order of ${\mathrm{ng}}/\sqrt {\mathrm{Hz}}$ (where g represents earth’s gravity) but they are typically high-cost and bulky. In this work the design of a bulk micromachined MEMS device exploiting non-linear buckling behaviour is described, aiming for ${\mathrm{ng}}/\sqrt {\mathrm{Hz}}$ resolution by maximising mechanical and capacitive sensitivity. High mechanical sensitivity is obtained through low structural stiffness. Near-zero stiffness is achieved through geometric design and large deformation into a region where the mechanism is statically balanced or neutrally stable. Moreover, the device has an integrated capacitive comb transducer and makes use of a high-resolution impedance readout ASIC. The sensitivity from displacement to a change in capacitance was maximised within the design and process boundaries given, by making use of a trench isolation technique and exploiting the large-displacement behaviour of the device. The measurement results demonstrate that the resonance frequency can be tuned from 8.7 Hz–18.7 Hz, depending on the process parameters and the tilt of the device. In this system, which combines an integrated capacitive transducer with a sensitivity of 2.55 aF/nm and an impedance readout chip, the theoretically achievable system resolution equals 17.02 ${\mathrm{ng}}/\sqrt {\mathrm{Hz}}$. The small size of the device and the use of integrated readout electronics allow for a wide range of practical applications for data collection aimed at the internet of things.

## Microelectromechanical systems: miniaturised device for high-resolution acceleration measurements

A micromechanical system (MEMS) has been developed that allows accurate measurement of acceleration at high-resolution levels. Commercially available gravimeters and seismometers can be used for high-resolution measurement of the earth’s acceleration, but they are typically very expensive and bulky. However, a team headed by Guo Qi Zhang at Delft University of Technology, Netherlands was able to design, simulate, fabricate and test a low-cost MEMS device with an integrated miniaturized high-sensitivity transducer for high-resolution acceleration measurements. The team was able to process and package its device by wirebonding it to a printed circuit board containing an application-specific integrated circuit. The authors believe that their extremely compact, low-power inertial sensor has considerable potential for a wide range of practical applications for data collection with the Internet of things.

## Introduction

Measurements of Earth’s gravitational field are becoming exceedingly important in building understanding of our planet’s behaviour, ranging from earth crust movement and volcanic eruptions to oil- and water pocket exploration and detection^1^. From the field of gravimetry, it is known that there are small variations in the gravitational force of the earth, typically caused by tides and ocean loading, local and global hydrology, volcanology, seismic activity and the presence of oil pockets^1^. These variations are in the order of nano-g (ng) to micro-g (µg). The collection of data from a dense grid of sensors detecting deviations in the earth gravitational force can be used to predict not only locations of oil- and water pockets in water-scarce environments, but also the movement of tectonic plates in the field of seismology and earthquake predictions. To accomplish this, gravimeters with high sensitivities in the order of ${\mathrm{ng}}/\sqrt {\mathrm{Hz}}$ are required for measuring the smallest changes in local gravitational acceleration^1^. However, commercially available high-end gravimeters and seismometers are typically large in form-factor, because rather extensive measurement techniques are used, i.e., falling masses. The underlying physical factors that complicate the development of relative and open-loop ${\mathrm{ng}}/\sqrt {\mathrm{Hz}}$ microelectromechanical system (MEMS)-based accelerometers, are the high mechanical sensitivity to be achieved and the displacement of a proof mass to be read out. High mechanical sensitivity in a relative MEMS-based accelerometer is directly related to the low resonance frequency of the structure, resulting in low stiffness and high-mass requirements. The magnitude of the proof mass is limited by the available chip area, especially as miniaturisation is typically one of the major advantages of MEMS technology. The low stiffness of the springs used is limited by process limitations, for example, the minimum resolution for etching. At last, combining a large proof mass with low stiffness springs inherently requires mechanical robustness and reliability to be considered.

Working out these challenges can thus result in a miniaturised, integrated, low-cost system capable of ${\mathrm{ng}}/\sqrt {\mathrm{Hz}}$ measurements that contributes to the recent advances in the Internet of Things (IoT)^2^.

In mass/spring-based accelerometer implementations, there is an inherent trade-off between operational bandwidth and resolution^3^. The bandwidth that is of most interest to the field of gravimetry ranges from the μHz up to several Hz, which corresponds to variations over a period of days down to intervals of seconds. Examples of such signals are secular deformation, seismic modes, and ocean tides^1^. The price paid by the increase in mechanical sensitivity, namely a lower bandwidth, is in line with the bandwidth requirements for the intended application field.

Recently, more research has been presented on the development of a miniaturised MEMS implementation of gravimeters by Middlemiss et al.^4^ Here, a MEMS gravimeter has been fabricated using bulk silicon micromachining for the design, which is based on the non-linear buckling of beams to enable high sensitivity reaching 40 ${\mathrm{\mu Gal}}/\sqrt {\mathrm{Hz}}$. Since 1 Gal equals 1 cm/s^2^, this sensitivity equals 40 ${\mathrm{ng}}/\sqrt {\mathrm{Hz}}$. The resonance frequency of the mass/beam system of this work is 2.3 Hz. The key feature of this work is the concept of exploiting non-linear buckling behaviour to obtain low stiffness and thus high mechanical sensitivity within a micromachined structure. However, the design does not further exploit the IC technology for the readout of the motion. Instead, this work employs an optical shadow sensor with bulky dimensions as an intermediate step to the electrical domain. An integrated capacitive transducer, in combination with an on-board integrated ASIC, would lead to a significant reduction in cost, facilitate further miniaturisation and significantly improve noise level performance.

Li et al.^5^ have fabricated a linear MEMS device that achieves 30 ${\mathrm{ng}}/\sqrt {\mathrm{Hz}}$ with a resonance frequency of 13.2 Hz. The competing value for the resolution was obtained by making use of a specially designed course/fine capacitive readout scheme. Nevertheless, the fabrication of this device is more complex, as the three dies need to be stacked to form the capacitive readout. This introduces fringe capacitances and large parasitic capacitances, resulting in high-noise contribution and lower sensitivity. The device uses linear springs, which have to be much stiffer than in the self-limiting non-linear design of this work to prevent catastrophic damage over the entire deflection range. In contrast to this work, the use of non-linear near-zero stiffness springs enables further improvement in the achievable resolution. Depending on the capacitive readout ASIC employed, a system resolution even beyond ${\mathrm{ng}}/\sqrt {\mathrm{Hz}}$ would be feasible. Another advantage of non-linear springs, which only locally show a near-zero stiffness, is that the dynamic range is not determined by the low spring constant alone. When an external force moves the non-linear spring out of its operating region, which is where it has minimum locally linearised stiffness, a deterministic response can still be measured despite the increase in stiffness.

Pike et al.^6,7^ presented a mass/spring-based accelerometer with resonance frequencies ranging from 0.1 Hz to 12 Hz. Impressive resolution figures are presented, 2${\mathrm{ng}}/\sqrt {\mathrm{Hz}}$^6^ and 0.25 ${\mathrm{ng}}/\sqrt {\mathrm{Hz}}$^7^. However, it should be mentioned that a closed-loop implementation is used, requiring more back-end signal processing. In addition, the layer-based transducer design featuring gold bars and micropositioned transducer strips implies more extensive processing compared with the objectives of this work. In terms of miniaturisation, the die size of 25 mm suggests room for improvement, especially for low-cost IoT applications. Compared with the work of Pike, this work specifically focusses on the development of an open-loop gravimeter exploiting standard CMOS compatible materials and technologies within a minimal die size.

The main drawback of state-of-the-art high-resolution accelerometers is thus the absence of a MEMS device capable of sub-Hz resonance frequency operation combined with an integrated readout scheme, while being fabricated in a CMOS compatible cleanroom and within a minimal die size. This combination would enable miniaturisation, (monolithic) integration with electronics and low-power operation. Furthermore, it enables direct transduction from the mechanical domain to the electrical domain instead of going via the optical domain.

The objective of this work is to design, fabricate and qualify an extremely compact ${\mathrm{ng}}/\sqrt {\mathrm{Hz}}$ open-loop type inertial sensor, fully based on MEMS and ASIC technology, which could even be monolithically integrated. The mass/beam system is designed for optimised sensitivity, torsional stiffness and integration with a capacitive transducer, which can be directly wire bonded to a PCB containing the readout ASIC.

To the best of our knowledge, this paper is the first in the literature featuring the combination of a non-linear MEMS device with an integrated bulk micromachined high-sensitivity capacitive transducer intended for high-resolution acceleration measurements.

**Fig. 1:**
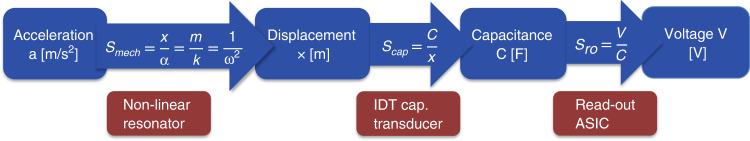
System overview of the gravimeter; note the different sensitivities between every physical domain

To achieve this objective, first a sensitivity analysis was performed over the different physical domains, as shown in Fig. 1. By maximising the sensitivity of each transduction, the noise of the subsequent stage in the sensor system is suppressed. By maximising both the mechanical sensitivity and the capacitive sensitivity, and choosing a high-resolution impedance readout scheme, a system resolution in the order of ${\mathrm{ng}}/\sqrt {\mathrm{Hz}}$ is aimed for, while keeping the operational bandwidth at 1 Hz.

The structure of the paper is as follows. In “Materials and Methods”, the concept of non-linear modelling is discussed, followed by the mechanical design aspects and implementation. In the second part of the section, the design of the capacitive transducer and the fabrication of the device is presented. In “Results and Discussion”, the measurements and noise analysis are described.

## Materials and methods

### Non-linear stiffness characteristic

**Fig. 2:**
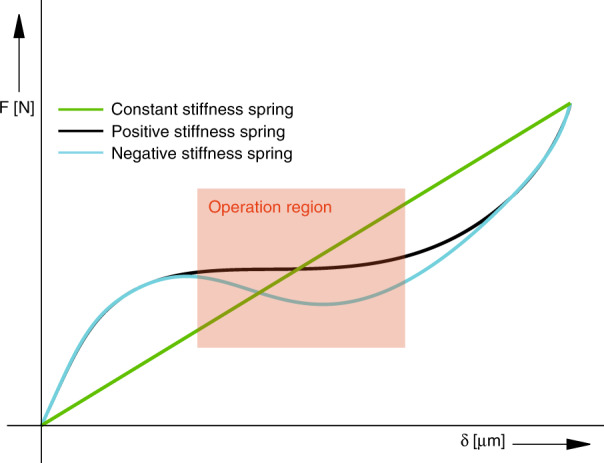
Mechanical beam force/displacement behaviour showing: constant high positive stiffness (green), low local positive stiffness (black) and low local negative stiffness (blue)

This work makes use of a non-linear effect which occurs in long slender beams, called buckling^8,9^. If the geometry of the beams is designed properly, this buckling can result in a localised region where spring-softening occurs. Consequently, for certain displacement values, the spring constant lowers, despite it being bounded by an increasing spring constant for displacements out of this range. This results in a local low-stiffness region in the force–displacement graph, as shown in Fig. 2.

A negative spring stiffness is not desired in this design, because multiple operating points would exist for the same reaction force applied causing snap-through to possibly occur, as is observed in bi-stable structures. Low stiffness of the beams results in high mechanical sensitivity, which scales down the noise contribution in the mechanical domain, such as Brownian motion or environmental noise, and the noise sources in the subsequent capacitive and electrical domains, as illustrated in Fig. 1.

### Design

**Fig. 3:**
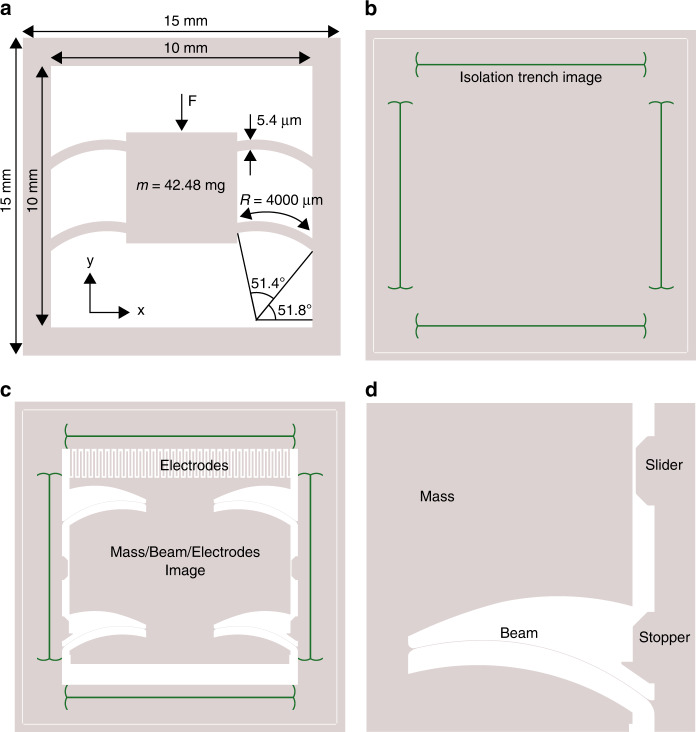
Geometrical design of the MEMS device. **a** Geometry of the mass/spring system, **b** trench isolation image, **c** Mechanical device with IDT electrodes and **d** designed slider and stopper structures

The design of the device structure is divided into modular parts: mass/beam structures including the electrodes and isolation trenches (Fig. 3b–d). Concretely this means that the device consists of a multi-image mask of which different versions can be combined during the lithography step of the fabrication process. Advantages of this are the more efficient use of reticle space and more processing flexibility, because different versions of the design parts are available. For example, two different trench widths are designed to ensure reliable filling of the isolation material later on.

The transducer structure consists of a proof mass, four slender beams and four capacitive comb electrodes, depending on the modular design options used. Different proof masses with different shapes and dimensions have been designed to be compatible with the different types of capacitive transducer structures. Three types of electrode dimensions have been designed to enable fine and coarse sensitivities. A minimum of four suspension beams are required to increase rotational and torsional stiffness. The beams are attached to the mass near the vertical axis of symmetry. This not only increases the rotational stiffness, but also makes the design more compact, because the beams require a certain length to combine the buckling effect with low spring stiffness.

The mass of the mass/beam system, being designed and fabricated planarly, can travel over a distance in the order of millimetres, which is relatively large for a 10 × 10 mm^2^ device. If the device is oriented vertically, the gravity force is in line with the displacement direction of the mass. The beams should be designed such that at this force they are pushed into the shape in which the buckling effect manifests itself and displays the constant stiffness region behaviour. Stopping structures have been designed to limit the deflection of the proof mass in case of high shocks to prevent self-damage.

### Geometric optimisation

The dimensions and design parameters of the mass and beam are described in Fig. 3 and have been determined iteratively using the known mechanical beam relation between them, originating from the beam theory of Timoschenko^10^. The parameters that were changed in the design to simultaneously obtain the correct resonance frequency and operating point at 1 g were: the sector angle of the beams, the rotation of the beams, the radius of the beams, the beam height and width and the value of the proof mass. The starting point is located at a beam height of 5 μm, which is the lowest beam height that can be fabricated. The beam radius and design angles determine the spacing each beam occupies, which affects the rotational stiffness. By reducing the radius to an optimum while increasing the mass width between two adjacent beams, the torsional stiffness increases. The spacing between the beams along with the radius are limited by the minimum device size. The resulting geometry of the interdigitated (IDT) design is included in Fig. 3a. The design of the mass/beam system was evaluated using the force–displacement relation obtained through a finite element analysis (FEM) in COMSOL. In the simulations, the length and thickness of the beam were 3588.4 μm and 5.4 μm, respectively. The radius and sector angles were 4000 μm and 0.8971 rad, respectively. The value range for the different parameters depends on the modular design.

**Fig. 4:**
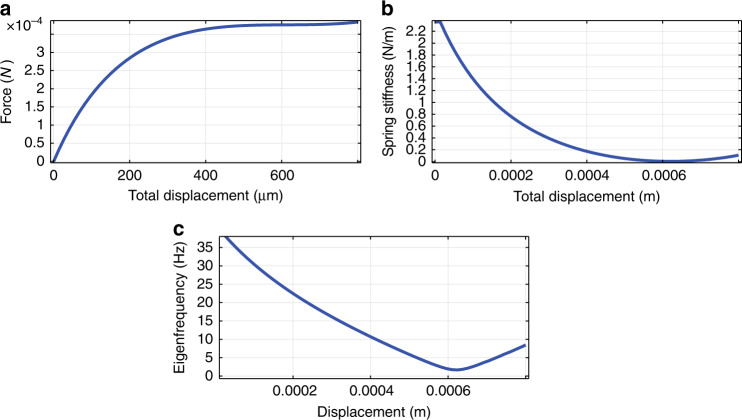
FEM simulation results on the non-linear spring design. **a** Force vs. deflection of the proof mass in the *y* direction, **b** stiffness as a function of displacement in the *y* direction, **c** resonance (Eigen) frequency as a function of displacement

The first simulation described covers the investigation of the force/displacement characteristic of the beams. The beams were modelled as 2D and 3D solid mechanics structures, with the inclusion of a non-linear solver. With the aid of prescribed displacement, the reaction forces were obtained. To transfer the reaction forces using the value of the proof mass, the *y* axis was scaled to plot the acceleration values according to Newton’s second law, as shown in Fig. 4a. The spring stiffness derived is the first-order derivative of the force–displacement relation. The result is included in Fig. 4b, where the lowest value of the spring stiffness corresponds to the operating point around the acceleration force, which corresponds to the nominal 1 g gravitational acceleration.

To ensure convergence in the mechanical simulations of the large-displacement non-linear design, a downward prescribed displacement is imposed on the proof mass in the physics section of the model. The displacement corresponding to the operating point then serves as the input to the resonance frequency analysis. The result is included in Fig. 4c, which shows the resonance frequency dependency on displacement with the lowest resonance frequency reaching 1.68 Hz, which corresponds to the first eigenmode in the *y* direction at the operating point as shown in Fig. 4c. The resonance frequency analysis further showed a second eigenmode beyond 107 Hz.

Despite the high aspect ratio and the layout of the geometry, deflections besides those in the *y* direction may cause a disturbance in the signal of interest owing to a finite spring stiffness in unwanted directions. By maximising the spring stiffness for the *x* and *z* directions, the risk of damage caused by plastic deformation instead of elastic deformation is minimised. This aspect is especially relevant in this work because the ratio of the mass-spring constant has been pushed to the limits of feasibility in order to obtain high mechanical sensitivity.

The FEM simulations conducted to obtain the spring constants in both the *x* and *z* directions were carried out in the same manner as for the *y* direction, except that the direction of the prescribed displacement was changed to the *x* and *z* direction, respectively. The proof mass is first set to the deflection corresponding to the operating point (1 g) in the *y* direction by a prescribed displacement imposed on the mass. Consequently, the reaction forces and spring stiffness in the other directions were determined from the operating point at 1 g with a deflection ~ 800 μm. The lowest stiffness in the 1 g operating point in the *z* direction is 2 × 10^4^ times higher than in the *y* direction. To give an estimation of the error caused by the out of plane displacement, a displacement of 50 μm in the *y* direction would result in a displacement of 3 nm at a tilting angle of 34°. The resulting capacitive error is then 1 × 10^−3^%. The stiffness in the *x* direction is initially similar to that in the *y* direction and increases rapidly beyond a value of 1.2 × 10^5^ N/m after 20 μm of displacement, owing to spring stiffening. A tilt with respect to the vertical axis can be applied to the device, resulting in a decreased *y* component acting on the proof mass. By doing so, the operating point and stiffness are changed accordingly. Thanks to the high stiffness in the *z* direction, the response of the sensor is not compromised by the out-of-plane force. The amount of deflection and thus the spring constant can be varied in this way, providing a trade-off between bandwidth and sensitivity.

### Design of the capacitive IDE transducer

While aiming for maximum capacitive sensitivity, the sensitivity in terms of ΔC was determined. For this analysis, a simplification was used, in which a linear addition of parallel plate equivalents is used. See also equation (1) below.

1 $${\mathrm{\Delta }}C_{tot} = \frac{{2N\varepsilon _0\varepsilon _rt\left( {y_0 - y} \right)}}{d}$$

In which *N* equals the number of fingers, *ε*_0_ the permittivity of vacuum, *ε*_r_ the relative permittivity, *t* the thickness of the structure (in this work wafer thickness), (*y*_0_ *−* *y)* the displacement in the *y* direction and d the gap between the electrodes. It can be concluded from equation (1) that the change in capacitance is maximised by maximising the number of fingers within the available design space and reducing the gap *d* between the IDT fingers.

The transducer is designed in such a way that it would be compatible with a bulk micromachined mass/beam system suspended in a frame of 10 × 10 mm^2^, which puts the first geometrical design constraint.

The lateral comb drive structure helps to reduce damping caused by air surrounding the structure. This can be explained by the couette flow, occurring for lateral motion, being much less dissipative compared with the squeeze film damping. Thanks to the main objective of measuring mechanical signals in the sub 20 Hz range, the fluid inside the gaps has enough time to displace but also prevents the air to act like a spring, which is storing and releasing energy at higher frequencies^11^. In addition, the damping can be further reduced by using vacuum packaging.

**Fig. 5:**
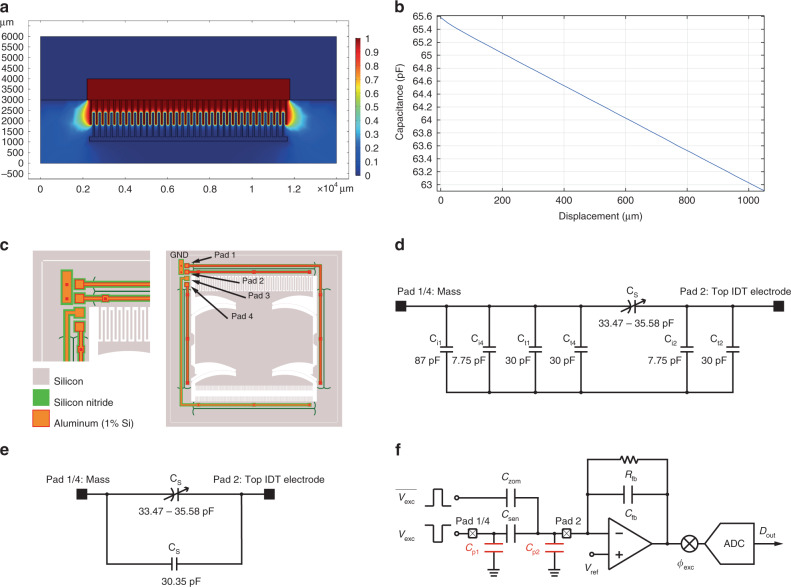
Capacitive FEM simulation results and the equivalent circuit of the MEMS device. **a** Potential distribution of the IDT and **b** Capacitance vs. displacement behaviour. **c** The device including trenches and interconnect. **d** Isolation trenches (denoted with subscript t) and interconnect (denoted with subscript i) parasitic capacitances associated with each connection pad. **e** Parasitic capacitance across the sense capacitor in case of floating GND. **f** Device and its readout

Capacitive simulations have been performed in order to investigate the electric field distribution and verify that the absolute value of the electric field does not exceed the dielectric strength of air. To simulate the capacitive response, an IDT electrode was placed across a second moving electrode. The capacitance/displacement behaviour is included in Fig. 5b. As can be concluded from this result, the capacitance varied from 35.58 pF to 33.47 pF. The values on the *y* axis however show larger values for the capacitance, because the trench for electric isolation is also included in this value. From this result, it can be concluded that the capacitive response as a function of deflection is highly linear with a large capacitive change per nm distance. Taking the first-order derivative of the capacitance versus displacement characteristic in the linear region yields a sensitivity of 2.55 (aF/nm) in the displacement region of interest.

The high degree of linearity in the capacitive response can be explained by the large number of fingers. The non-linear effect of the fringing E-field on either side of the electrode array is suppressed by the large contribution to the total capacitance of the normal components of the field between the parallel ‘fingers’. To illustrate this, the electric field distribution is included in Fig. 5a, where the fringing E-field (boundary) effects are visible. In addition, it can be noted that the aspect ratio of the structure is high: the width of each finger equals only 70 µm, whereas the thickness of the structure equals wafer thickness of 300 µm. The large width of each finger suppresses the non-linear effect caused by fringing fields at the beginning and end of the IDE finger array, while the large height of each IDE finger suppresses the fringing fields on front- and backside of the wafer.

### Parasitic capacitances

The IDT structure is fabricated from the same substrate as the mass/beam system. Therefore, an electrical isolation was required and was realised using trenches filled with a dielectric. As a result, parasitic capacitances between the main frame and the IDT electrode regions inevitably arise. Additional parasitic capacitances are caused by the metal traces connecting the different potential regions with the probe pads. Both sources of parasitic capacitances are illustrated in Fig. 5c.

The dielectric material is silicon nitride (Si_3_N_4_) for both the trenches and interconnect parasitics. To analyse the parasitic capacitances an equivalent circuit model was made, based on the actual way of interfacing the sensor. This equivalent circuit model is included in Fig. 5d, illustrating the capacitances as these will appear across the connection pads. The subscript including an ‘i’ denotes a parasitic caused by the metal trace, whereas a subscript including ‘t’ denotes a parasitic caused by an isolation trench.

All the parasitics on either side of the sensor capacitance C_s_ can be simplified to one parallel capacitor by omitting the ground connection, reducing the total parasitic capacitance. The equivalent circuit in this case is included in Fig. 5e. However, in this case, the parasitic capacitance will be in parallel to the sense capacitance. This effect is undesired, because charge redistribution from the measurement signal over the parallel capacitor occurs. This would result in a worse system performance compared with the parasitic capacitance being connected to ground, as shown in Fig. 5d. The best way of connecting the sensor is summarised by Fig. 5f, thus including the connected ground.

To operate the capacitive sensor, a square-wave signal *V*_exc_ is applied, at a frequency higher than the corner frequency defined by *R*_fb_ and *C*_fb_. Simultaneously, the zoom-in capacitor, *C*_zom_, is excited by a signal, inversed *V*_exc_, to cancel the baseline capacitance, thus maximising the use of the capacitance to voltage converter’s (CVC) available dynamic range. The sensing signal induced capacitance changes modulate the excitation current. The resulting amplitude modulated AM signal is then amplified and demodulated back to in-band. As the modulated sensing signal is processed by the amplifier in high frequency where the 1/f noise is not dominating, the noise performance of the CVC gets improved. Finally, the voltage signal is digitised by an analogue-to-digital converter (ADC).

### Fabrication considerations and geometry

The displacement is read out using an IDT capacitive structure etched in the silicon substrate along with the mass/beam system. Since the electrodes are made from the bulk silicon, both electrodes of this transducer consist of low-ohmic silicon, which requires electrically isolated regions to facilitate the readout process. The target chip area of 10 × 10 mm^2^ needs to be taken into account when designing the capacitive transducer for maximum sensitivity, which restricts for example the maximum number of fingers in the IDT array.

The IDT has been designed with finger widths and air gaps of 70 μm and a capacitive sensitivity of 2.52 × 10^−9^ F/m, which corresponds to 2.52 aF/nm. The overlap between the fingers starts at 1040 μm and decreases for increasing deflection.

**Fig. 6:**
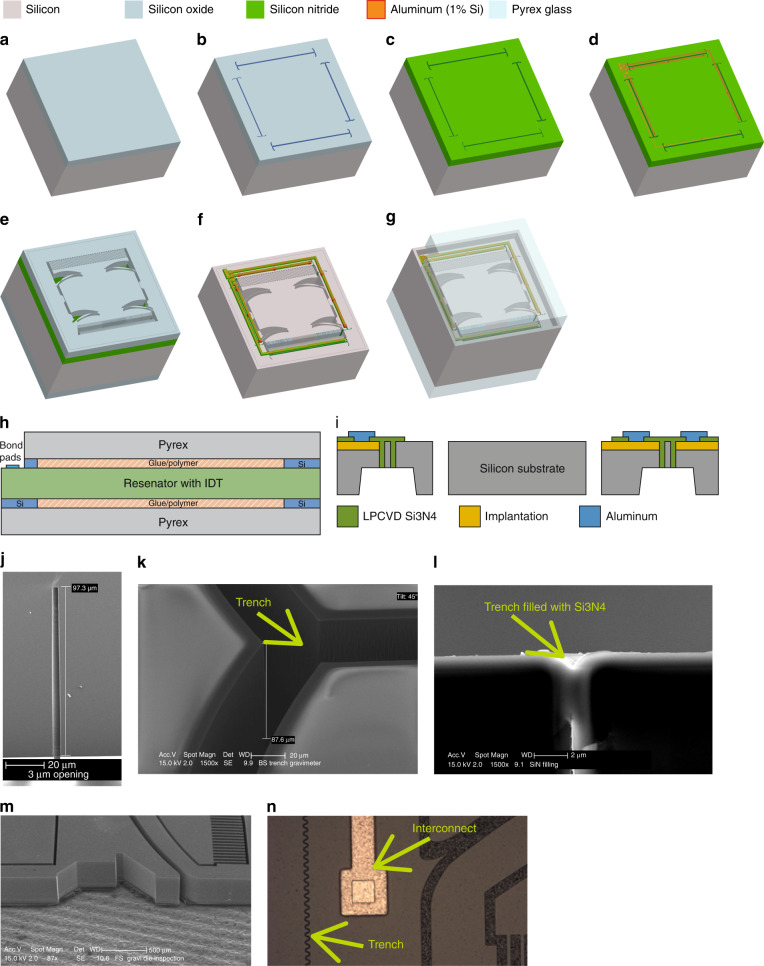
Process flow of the MEMS device. **a** Hard mask, **b** trench isolation patterning, **c** trench filling with Si_3_N_4_, **d** metal interconnects on the front, **e** back hard mask with mass/beam patterns, **f** released device, **g**, **h** final packaged device, **i** the schematic cross section showing the trench isolation technique, **j** etched trench 97.3 µm deep and 3 µm wide, **k** top view of etched trench, **l** etched trench filled with SiN as the isolator, **m** DRIE etched structures before release, **n** a microscope image in which the metal trace and the isolation trench can be seen

Because both electrodes of the capacitive transducer are bulk micromachined from the same substrate, electrical isolation is required. This is done by etching trenches ~100 μm deep and filling these with silicon nitride for isolation and mechanical connection purposes. Figure 6j shows a SEM image of the etched trench with a depth of 97.3 μm and width of 3 μm. Figure 6k, l show the SEM images of the trench before and after being filled with Si_3_N_4_ as an isolator.

The spring stiffness in unwanted directions (*x* and *z* direction) is at least two orders of magnitude larger with respect to the *y* direction. Additional structures are included to limit large translation and rotations and to prevent electrodes from touching. Stopping structures are included in the design to prevent the electrodes from short circuiting in the lateral direction as a result of large forces. Figure 3d shows these structures.

### Device fabrication

The fabrication of the inertial transducer was performed in a CMOS compatible cleanroom and using MEMS microfabrication starting with 4-inch p-type silicon wafers (300 μm thick) with a resistivity of 10 Ωcm.

First, electrical isolation steps for separating the electrodes from the bulk were followed. The steps consisted of (1) etching silicon from the frontside through trench formation, (2) filling the trenches using low pressure chemical vapour deposition (LPCVD) silicon nitride (Si_3_N_4_) for electrical isolation and mechanical adhesion and (3) etching the remaining silicon from the backside to complete the electrical isolation. The latter was achieved after the definition of the mass/beam structures in the silicon. After every pattern etching step, a cleaning procedure was performed to remove any organic material such as the photoresist or polymer used for wall passivation in the deep reactive ion etch (DRIE) etching process.

Figure 6a–c show the first electrical isolation step where a silicon oxide (SiO_2_) masking layer was deposited on the frontside of the substrate, patterned using standard lithography and etched using reactive ion etching (RIE). Hereafter, a DRIE step was performed to etch 100 μm deep trenches. Subsequently, the substrate was cleaned after which the SiO_2_-masking layer was then removed using 1:7 hydrofluoric acid (HF) wet etching. The etched trenches were then filled with the isolation material in three steps. Each of these steps consisted of a deposition followed by a planarisation without masking until the substrate was exposed. This method helps in minimising the risk of voids forming and in achieving a more uniform surface, which is an advantage during the later contact metallisation step. Prior to the last deposition step a boron implantation step was performed for ohmic contact formation with a dose of 10^15^ cm^−3^ at an energy of 20 keV. Because the Si_3_N_4_ layer is also used as isolation layer between metal interconnect and substrate, after the third deposition, no subsequent etching was performed, as illustrated by Fig. 6c. The annealing step of the implantation was then carried out simultaneously with the last LPCVD deposition step at 800 °C. To connect the device to the outer world, contact openings were etched into the Si_3_N_4_ layer. Subsequently a 1 μm aluminium (Al) layer with 1% Si was deposited for metal interconnects and was patterned using standard lithography and inductively coupled plasma (ICP) etching for metals (Fig. 6d).

**Fig. 7:**
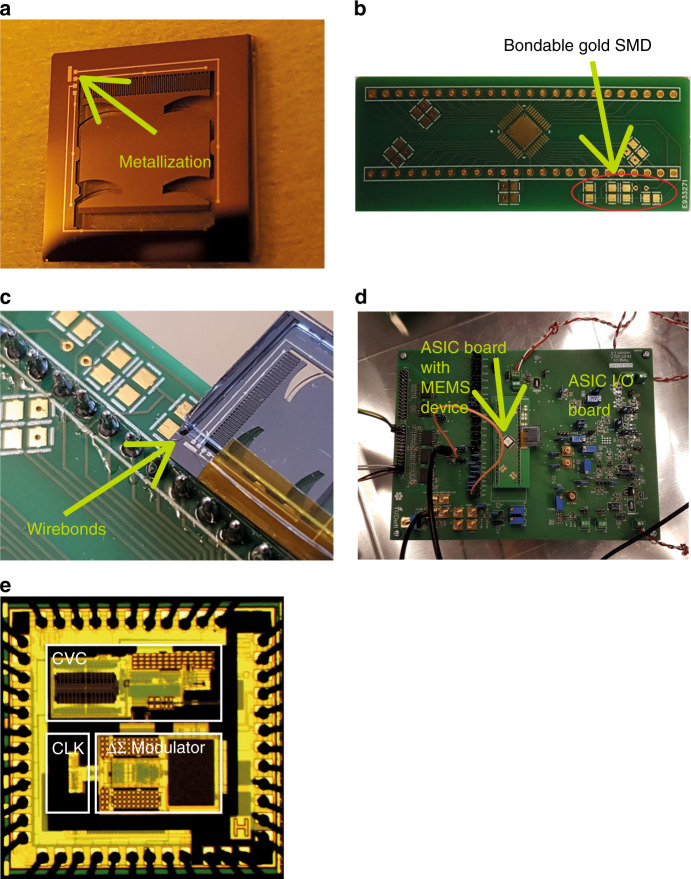
Packaging and integration of the fabricated MEMS device with the readout system PCB and ASIC. **a** Final device after release, **b** interconnect PCB on which the ASIC is situated; the bondable gold SMD pads (circle) are used to wirebond the mass/beam system die on, **c** UV glued and wire-bonded accelerometer on top of the daughter PCB and, **d** daughter PCB in the measurement setup, along with the metal box to shield the setup from external EMI interference, **e** ASIC chip

To complete the electrical isolation and define the mass/beam structures, two DRIE-etching steps were performed. The steps consisted of the deposition of SiO_2_-masking layers at the frontside and backside, respectively. Starting with the backside, the masking layer was patterned with isolation trenches to complete the electrical isolation. This was achieved using standard lithography followed by RIE- and DRIE-etching steps. Similarly, the frontside was patterned with the mass/beam structures. The DRIE-etching step was performed until a through-wafer etch was achieved. Figure 6e shows the resulting structure. The last steps were the removal of any unwanted materials such as the passivation layer by an oxygen plasma, the Al-capping layer by ICP etching, and the SiO_2_ hard masks by vapour HF etching. After the vapour etching, the devices were released manually through vibrations during which the released halo-structures detached from the substrate (Fig. 6f). Figure 7a shows a picture of the device as it was realised using this procedure.

### Packaging and integration of the readout ASIC

After the dies were released in the cleanroom, packaging was needed to protect the proof mass from contaminants that could block the IDT structure. The packaging shields the mass/beam system from air flow, which may damage the device. Because the device is based on the concept of buckling using large-displacement behaviour, it is desired to use a packaging solution that allows visual inspection.

The silicon die is therefore packaged on both sides using glass dies fabricated from a Pyrex wafer, as illustrated in Fig. 6g. Because the sensor is bulk micromachined, the moving parts have the same thickness as the rest of the die, in this case the frame. To allow the mass to move freely within the glass-silicon-glass stack, silicon spacers are used to have a controlled and known separation between the mass/beam system and the outer glass dies as shown in Fig. 6h. The stack of glass outer dies, spacers and MEMS-chip is glued together by ultraviolet curable Norland Optical Adhesive.

The readout ASIC has a measurement range of 80 pF. The readout ASIC has a 10 pF on-chip capacitance and a digital-to-analogue converter to compensate the baseline and the parasitic capacitance of the sensor, such as the ones originating from the isolation trenches and interconnecting metal traces.

To minimise the parasitic capacitance or inductance in the packaging scheme further, the sensor has been directly wire bonded onto a PCB housing the ASIC and I/O to the DAQ system. These wirebonds will introduce (minimal) parasitic capacitance and inductance, but as the variation in C around an equilibrium position is of main interest, these parasitics will not affect the measurement. The PCB has numerous options for capacitive readout, such as capacitance offset compensation and connections for bridge readout. The PCB is shown in Fig. 7b prior to soldering the ASIC and wirebonding the accelerometer to it. Figure 7c shows the wire-bonded accelerometer. A picture of the ‘daughter PCB’ with the readout ASIC and die embedded in the main PCB is included in Fig. 7d. The ASIC chip is shown in Fig. 7e.

## Results and discussion

The devices were tested at room temperature by measuring the capacitive response at different tilt angles. The measurement results are presented in the time and frequency domains and used to characterise the fabricated devices for their resonance frequencies and corresponding sensitivities. The presented concept can be used as starting point for a future development of a closed-loop implementation^12^. When doing so, the measurement range can be further extended, while maintaining high resolution. As such a design would require actuation, this will be only considered for future iterations.

### Time domain measurements

The devices were tested by clamping the PCB onto a manual tilting stage and increasing the tilt from a horizontal starting position. Accordingly Earth’s gravitational acceleration of 1 g is scaled with the sine of the tilt angle. As a result, bringing the proof mass to the ‘biasing point’ where low spring stiffness occurs can be performed in a highly controllable manner.

As a test signal, the mechanical shaker from Brüel & Kjaer (model 4810) was used, exciting a sinusoidal signal in the frequency range of the first resonance frequency of the devices. This range was determined by observing the displacement amplitude of the proof mass while manually varying the excitation frequency. When the excitation signal passes the resonance frequency, resonance in the mass beam system is triggered and the movement of the proof mass shows a periodic behaviour.

The readout chip makes use of a Sigma-Delta-based ADC with a sample rate of 2 MS/s. The digital bitstream is decimated by averaging a specific number of bits, which returns to the concept of over-sampled data converters. By choosing the decimation factor, there will be a trade-off between the sample rate in the time domain and the resolution per sample. The decimation factor used here equals 10^4^. Subsequently MATLAB was used to further analyse the measurement data.

**Fig. 8:**
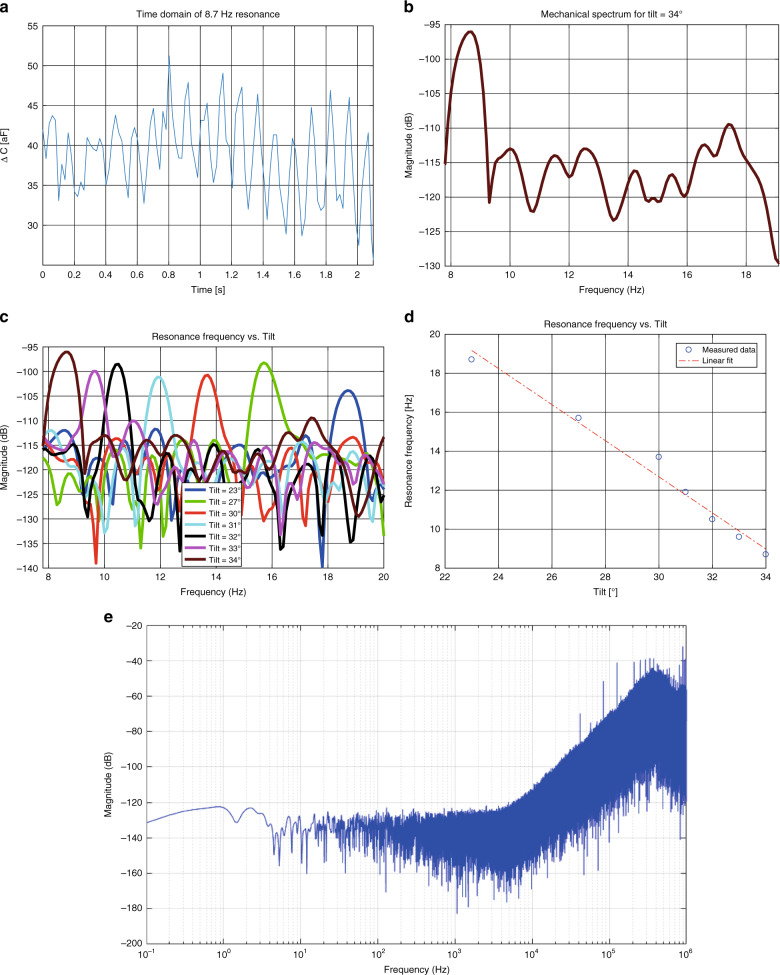
Measurements results in time and frequency domains and the noise floor of the CVC. **a** Measurement during resonance of the device at a tilt angle of 34° (time domain), **b** measurement during resonance of the device at a tilt angle of 34° (frequency domain), clearly showing the resonance tone at 8.7 Hz, **c** mechanical spectrum of the device for tilts ranging from 23° to 34°, **d** resonance frequency as a function of the tilt, and **e** noise floor of the capacitive readout scheme

The first measurement result is illustrated in Fig. 8a, showing two seconds from a 5 s interval during a frequency sweep from 5 to 9 Hz in the time domain. Despite the presence of noise and drift, the periodic signal of the resonance behaviour can be clearly recognised. The noise was probably caused by interference from the surroundings, such as 50 Hz disturbance or electromagnetic Interference from the measurement equipment to the exposed wirebonds. When determining the average period of the time domain signal, it can be concluded that the frequency corresponds to the 8.7 Hz resonance frequency value.

### Frequency domain: tilt measurements

After the time domain signal was analysed based on the discrete sample, the PSD of the signal was derived by applying an FFT operation on the data. For the analysis of the mechanical spectrum of the sensor chip, the effect of different tilt angles was investigated in this series of measurements. The tilt of the sensor was varied from 23° to 34°. For every measurement, a sweep of the mechanical excitation signal was performed while the capacitance was simultaneously sampled. The spectrum of the measurement results is included in Fig. 8b, c. Here, the resonance frequency can be clearly distinguished from the noise level, and is decreasing with increasing tilt angle. This response on the tilt angle can be expected from the combination of a fixed proof mass in combination with a softening spring. With increasing deflection, which is in turn caused by a larger tilt, a larger value for the gravitational force is working on the proof mass. The capacitive transducer is designed for device operation in line with the acceleration under test. As we apply a tilt in this series of measurements, it can be expected that, owing to the finite stiffness in the ‘out-of-plane’ direction, the magnitude of the capacitive response is influenced. The tilt measurements are used to identify the location of the resonance frequencies. In a final application, the tilt will be 90°, resulting in no ‘out-of-plane’ movement.

The tilt measurements show that the resonance frequency of the sensor is tuneable by varying the tilt. As a result, the mechanical sensitivity, being a direct function of the stiffness and thus implicitly related to the resonance frequency, of the sensor is also tuneable. The lowest resonance frequency obtained with the fabricated sensor chip is 8.7 Hz with a tilt angle of 34°.

To further investigate the relation between the tilt angle and the resonance frequency, both figures are plotted against each other in Fig. 8d. The plot in this figure was obtained by taking the maximum values from Fig. 8c. It can be concluded from this result that the resonance frequency is highly sensitive to the tilt angle. Every 1° of increased angle results in a drop in the resonance frequency by ~1 Hz, which is significant in this low frequency range. The degree of linearity can be explained by the simulation result included in Fig. 4c. The resonance frequency equals the square root of the stiffness over the mass, the first one being simulated to show a linear behaviour with deflection and the second one being constant. This measurement shows again that by applying a tilt to the sensor chip, a trade-off can be carried out over the dynamic range, in terms of the operational bandwidth and measurement range, and mechanical sensitivity.

### Sensitivity analysis of the measurement system

The minimum detectable acceleration is determined by transferring back the total noise in the measurement system to the input. The two noise sources considered in a capacitive accelerometer are the thermal–mechanical noise and the noise originating from the analogue front-end electronics^13^. Determining the input-referred resolution is done by dividing each noise contribution by the sensitivities applicable, in this case the mechanical- and capacitive sensitivities. The first one is dictated by the resonance frequency, whereas the second one is taken from the design of the IDT structure. The thermal–mechanical noise comes back to the derivation of the fluctuation–dissipation theorem^13^, which basically means that the damping mechanism in the system produces a noise density based on the damping constant and temperature. In the design of this sensor, no additional damping was added, which means that the mass can move freely within its structure. The only damping available is that of the intrinsic damping of the material and that of the surrounding air. To analyse the thermal–mechanical noise, also called Brownian motion noise, the following derivation, which is already translated from force noise via displacement noise to acceleration noise, was used. The formula is described by Senturia^14^.

2 $$a_{\mathrm{n}} = \sqrt {\frac{{4k_BT\,{\mathrm{\omega }}_0}}{{mQ}}}$$

where *k*_B_ is the Boltzmann constant, *T* the absolute temperature, *ω*_0_ the angular resonance frequency, *m* the mass of the proof mass and *Q* the quality factor, which is in this case determined with:

3 $$Q = \frac{{f_0}}{{f_2 - f_1}}$$

for which *f*_0_ is the resonance frequency, and *f*_1_ and *f*_2_ are the − 3 dB frequencies on either side of the tone.

Using the lowest resonance frequency obtained, 8.7 Hz, a mechanical quality factor for the structure of *Q* = 12.43 is yielded and an input-referred system resolution value is obtained for ${\mathit{a}}_{\mathrm{n}} = 4.03\,{\mathrm{ng}}/\sqrt {\mathrm{Hz}}$. Packaging of the resonator under vacuum will improve the quality factor, by reducing the damping by the (squeezed) air around the moving proof mass, in turn reducing the thermal noise contribution by a lower damping coefficient *b*.

The resolution of the capacitive readout scheme, including the current-to-voltage converter (IV-converter) and ADC, within a BW of 20 Hz is $0.137\,{\mathrm{aF}}/\sqrt {\mathrm{Hz}}$ (ASIC specification). With a capacitive sensitivity of 2.52 aF/nm (design), this corresponds to a displacement noise of $0.054\,{\mathrm{nm}}/\sqrt {\mathrm{Hz}}$. Using the lowest resonance frequency measured and the second derivative of the harmonic oscillation, the mechanical sensitivity equals:

4 $$S_{mech} = \frac{{x\left( m \right)}}{{a\left( {ms^{ - 2}} \right)}} = \frac{1}{{\left( {2\pi 8.7} \right)^2}} = 3.35.10^{ - 4}\frac{m}{{ms^{ - 2}}}$$

The resulting resolution in terms of acceleration is $1.62 \cdot 10^{ - 7}\,{\mathrm{ms}^{ - 2}}/\sqrt {\mathrm{Hz}}$. By normalising this to values of a gravitational constant, a value of $16.54\,{\mathrm{ng}}/\sqrt {\mathrm{Hz}}$ is obtained.

Assuming that the noise contribution from the thermal–mechanical noise and the electronic noise from the front-end are uncorrelated, both input-referred noise power sources can be added and taking the square root, yielding an input referred system resolution of $17.02\,{\mathrm{ng}}/\sqrt {\mathrm{Hz}}$.

## Conclusion

In this work, a bulk micromachined mass/beam system has been designed, simulated, fabricated, tested and characterised. The device was processed, packaged using glass dies and wire bonded to a PCB containing a readout ASIC. The realised MEMS device with an integrated capacitive transducer was packaged and directly wire bonded to a high-resolution impedance readout system forming a measurement system. By using the non-linear force/displacement characteristic of buckling beams, an operating point has been created with extremely low stiffness. The vertical displacement of the proof mass is readout by a capacitive transducer, which is integrated into the same bulk micromachined silicon die. This is possible thanks to the trench isolation technique, which electrically separates both parts of the silicon bulk while keeping lithographic lateral resolution. The presented concept shows the potential for an integrated platform of an extremely compact MEMS + ASIC gravimeter, both on the package level, or even a monolithically integrated CMOS in the MEMS device.

When combining the lowest resonance frequency of 8.7 Hz, the capacitive sensitivity and the experimentally characterised noise floor of the readout chip, the theoretically obtainable system resolution equals $17.02\,{\mathrm{ng}}/\sqrt {\mathrm{Hz}}$. This figure for the theoretically obtainable resolution shows that the integration of the non-linear buckling-based compliant mass/beam system with the bulk micromachined capacitive transducer yields a concept for a high-resolution MEMS-based accelerometer. The proof of concept is promising for future iterations to yield a low-power, miniaturised and integrated MEMS solution for high-resolution acceleration measurements. Although stoppers have been designed to make the device robust against shocks, robustness testing and more extensive packaging design is open for future research.

## Supplementary information

Supplementary information

## Data Availability

The simulation files and data sets generated and analysed in this work are available from the authors on request.
