# Supplementary material for: High-resolution MEMS inertial sensor combining large-displacement buckling behaviour with integrated capacitive readout
Source: Microsyst Nanoeng. 2019 Dec 16;5:60. doi: 10.1038/s41378-019-0105-y (PMC8433333; doi:10.1038/s41378-019-0105-y)
Supplement: Supplementary file 1 — Supplementary information [file 41378_2019_105_MOESM1_ESM.pdf]

Supplementary information

Processing

At the exact location of the trenches a cavity is etched on the backside of the wafer to remove the conductive silicon and isolate both parts of the device. The etching windows for the isolation trenches have been made ($3 - 5\ \mu m$ wide) to enable different filling profiles. This is an important step to overcome the dependency of one specific filling material for good step coverage and mechanical stability during fabrication. Moreover, the filling material and the width of the etching windows have a direct impact on the parasitic capacitances to ground, which determines the readout chip requirements. The isolation trenches have a serpentine-like structure to increase the reliability of mechanical binding between the isolated islands and the bulk as a whole.

To ensure good coupling between the tip and the substrate during wire bonding, ultra-thin double-sided μm tape is used. Normal tape would give too much damping which would inhibit the bonding process.
